# Transcription of GABA_A_ receptor subunits in circulating monocytes and association to emotional brain function in premenstrual dysphoric disorder

**DOI:** 10.1038/s41398-025-03465-6

**Published:** 2025-07-23

**Authors:** Louise Stiernman, Erika Comasco, Maja Johansson, Marie Bixo

**Affiliations:** 1 Department of Clinical Sciences, Umeå University, Umeå, Sweden; 2 Umeå Center for Brain Imaging (UFBI), Umeå University, Umeå, Sweden; 3 Department of Women’s and Children’s Health, Science for Life Laboratory, Uppsala University, Uppsala, Sweden

**Keywords:** Psychiatric disorders, Molecular neuroscience

## Abstract

Premenstrual dysphoric disorder (PMDD) has been hypothesized to be related to an altered sensitivity of the γ-aminobutyric acid type A (GABA_A_) receptor to progesterone-derived neurosteroids. GABA_A_ receptor sensitivity to neurosteroid-modulation is dependent on its subunit composition. In the present study, we used quantitative reverse transcription polymerase chain reactions (RT-qPCR) to compare messenger ribonucleic acid (mRNA) expression of GABA_A_ receptor subunits in peripheral mononuclear cells (PBMCs) across the menstrual cycle in 29 women with PMDD and 27 controls. We related mRNA subunit expression to serum levels of neurosteroids and to functional activation of the amygdala, a key brain region involved in emotion generation, measured using functional magnetic resonance imaging (fMRI). Women with PMDD had lower mRNA expression of the delta GABA_A_ receptor subunit during the symptomatic, luteal phase (compared to the asymptomatic, follicular phase) of the menstrual cycle. Lower delta mRNA expression was related to higher amygdala activation in PMDD women. GABA_A_ receptors incorporating the delta subunit are especially sensitive to neurosteroid modulation. It is possible that the mood symptoms of PMDD are mediated by an inability to effectively adjust the expression of this receptor type in response to neurosteroid fluctuations, leading to dysregulation GABAergic tone and increased activity in emotion-generating brain circuits.

## Introduction

Premenstrual dysphoric disorder (PMDD) affects 2–6% of menstruating women and is associated with significant impairment and lowered quality of life [1, 2]. The disorder is characterized by debilitating mood symptoms in the luteal (premenstrual) phase of the menstrual cycle. Ovulation, and the subsequent production of progesterone characterizing the luteal phase, is a prerequisite for mood deterioration in PMDD [3]. One hypothesis for PMDD is that it may be a disorder of altered sensitivity to progesterone’s neurosteroid metabolites - such as allopregnanolone (ALLO) - which are potent positive modulators of the γ-aminobutyric acid type A (GABA_A_) receptor [4].

The GABA_A_R is composed of five subunits from a possible 19: α1–6, β1–3, γ1–3, ρ1–3, δ, ε, π, and θ [5]. The pharmacodynamic properties of the receptor, including its sensitivity to neurosteroids, are determined by its subunit composition [6, 7]. Neural expression of α, β, γ and δ subunits has been shown in mice to be influenced by the estrous cycle, stress induction and administration of neurosteroids and other GABA_A_ modulating drugs [8–13]. Interestingly, α4 and δ-containing receptors are highly sensitive to ALLO, and are upregulated by ALLO withdrawal after chronic exposure, and paradoxically, also by acute ALLO exposure [11, 14, 15]. ALLO-mediated increases in expression of δ-containing receptors during late diestrus (analogous to the human late-luteal phase) in the mouse have been shown to mediate lowered neuronal excitability and diminished anxiety behaviors [12]. Knock-out (δ−/δ− and δ+/δ−) mice show depressive behaviors upon withdrawal from long-term progesterone/ALLO exposure (i.e. pregnancy), which are alleviated by a specific δ-receptor agonist in (δ+/δ−) mice [16]. In summary, these findings suggest that GABA_A_Rs, and δ-GABA_A_Rs in particular, are subject to dynamic regulation in conditions of fluctuating ALLO levels. Efficient regulation of these receptors seems to be crucial to maintain physiological neural excitability and euthymic mood. Indirect evidence for altered regulation of GABA_A_Rs in PMDD across the menstrual cycle comes from pharmacological challenge studies, which have shown that women with PMDD have abnormal pharmacodynamic responses to GABA_A_-active substances, including benzodiazepines [17, 18], ethanol [19], and ALLO [20]. Furthermore, we recently showed that levels of ALLO in relation to its 3β-epimer isoallopregnanolone (ISO) (which specifically antagonizes ALLO’s effect at the GABA_A_R) are associated with differential activation of the amygdala, a brain region commonly implicated in the psychopathology of mood disorders [21]. However, studies that directly quantify GABA_A_R subunit expression and its influence on neural activity and premenstrual mood in PMDD are lacking.

Methods for studying changes in specific subunit composition of the GABA_A_R in the living human brain are limited. However, GABA_A_Rs are expressed elsewhere in the body, including in circulating cells of the immune systems [22]. The expression of GABA_A_R subunits in peripheral blood mononuclear cells (PBMCs) has previously been shown to be modulated by pregnancy and depression in women [23]. However, GABA_A_R subunit expression has never been analyzed across the human menstrual cycle. PBMCs may thus offer a unique opportunity to study GABA_A_R plastic changes in response to altered ovarian hormone levels in PMDD.

We aimed to measure changes in mRNA expression of GABA_A_R subunits in PBMCs of women with PMDD and asymptomatic controls across the menstrual cycle. Furthermore, we investigated whether differential GABA_A_R subunit expression in the symptomatic, luteal phase was related to amygdala reactivity during an emotional task, determined using functional magnetic resonance imaging (fMRI). We also related differential GABA_A_R subunit expression to premenstrual symptom severity, and to serum levels of ALLO and ISO.

## Methods

The sample of women included in this study has been described in one of our previous publications, which investigated emotion-induced brain activity using functional magnetic resonance imaging (fMRI) [21]. Participant recruitment, eligibility criteria, PMDD diagnostic procedures and steroid quantification are thus identical to the ones described earlier [21]. The same is true for MRI acquisition procedures, preprocessing and first-level (individual-level) analyses of fMRI data. In the present study, we provide only brief descriptions of the methods that have previously been described [21].

### Subjects

Twenty-nine women with PMDD and 27 asymptomatic controls were included. General inclusion criteria were: ages 18–45 years, regular menstrual cycles (25–31 days), use of non-hormonal contraceptives, and oral and written consent. Exclusion criteria included: significant ongoing psychiatric or somatic condition, current use of steroid hormone or psychotropic medication (including herbal remedies), drug or alcohol abuse, and pregnancy. Minimum wash-out periods of three months for psychotropics drugs (e.g. selective serotonin inhibitors) and one month for hormonal contraceptives were required prior to entering the study. In addition, a minimum “wash-out” period of three months and resuming of normal menstrual cycling was required if a woman had newly given birth or breastfed a child. Potential participants (both PMDD and control groups) were screened for past and current psychiatric conditions as well as drug/alcohol abuse in the past year using the Mini international Neuropsychiatric Interview (MINI) questionnaire [24]. Due to their high prevalence, eating disorders and depressive episodes were accepted, given that they had been in complete remission for a minimum of two years. In addition, all participants provided daily ratings of mood, psychological and physical symptoms prior to inclusion using the Daily Record of Severity of Problems (DRSP) for a minimum of two menstrual cycles. The DRSP is a reliable and validated tool for PMDD diagnosis [25], and was implemented via a web platform.

### PMDD diagnosis

PMDD was confirmed using DRSP ratings and the algorithm formulated by Endicott et al [25]. Briefly, criteria had to be met for two menstrual cycles and were as follows: (1) in the mid-follicular phase (days +6–+10), no mean symptom scores greater than mild; (2) in the late-luteal phase (days −5–−1), moderate symptoms on at least one mood item, and five symptoms overall; (3) symptoms in the late-luteal phase impacted functioning in daily life.

Control women were included if they had no average symptoms greater than mild during either the follicular or luteal phase of the menstrual cycle.

### Study design and sample collection

Two study visits occurred once in the mid-follicular phase (days +5–+11) and once in the late-luteal phase (days −8–−1). Menstrual cycles were tracked for each participant during screening and throughout the study period to provide better estimates of average menstrual cycle length. We estimated that ovulation would occur 14 days prior to the next onset of menses and scheduled study sessions thereafter. Ovulation was confirmed through serum progesterone levels as well as self-reports of menses onset. Luteal phase progesterone concentrations were required to fall within 2 standard deviations (SD) of the standardized progesterone curve [26] for the menstrual cycle day during which the study visit occurred. On each visit, participants underwent fMRI scanning. Prior to scanning, serum samples were drawn by antecubital puncture in BD Vacutainer CPT cell preparation tubes with added sodium heparin (Becton Dickinson) and immediately centrifuged at 1500 *g* for 15 min at room temperature. Half of the serum was aliquoted to sterile sodium-heparin containing cryogenic vials (Avantor, VWR) and frozen at −80 °C within 30 min for subsequent quantification of progesterone ISO, and ALLO levels. The remaining plasma and buffy coat were kept on ice until PBMC isolation within 15 min.

#### PBMC separation

RPMI-1640 medium (Sigma Aldrich) was added to the cell suspensions containing buffy coat and plasma and centrifuged at 250 *g* for 10 min at room temperature. The resultant pellet was resuspended in fetal bovine serum (FBS) (Sigma Aldrich) to reach a concentration of 1 × 107 cells/ml. The same volume of cell freezing medium, composed of 75% heat-inactived FBS and 25% dimethylsulfoxide (DMSO) (Sigma Aldrich), was added drop-by-drop to the resuspended pellet. The samples were then aliquoted to Nunc cryogenic vials (Sigma Aldrich) and placed in a CoolCell freezing container (Sigma Aldrich) to ensure a slow and gradual cooling to −80 °C.

### Total RNA isolation and real-time quantitative polymerase chain reaction (qPCR)

Total RNA was isolated using RNeasy Mini Kit (Qiagen) and quantified with Nanodrop (Thermo Fisher Scientific). Total RNA (500 ng) was reverse-transcribed into cDNA in a 25 µl reaction mixture using High Capacity cDNA Reverse Transcriptase Kit with RNase Inhibitor (Applied Biosystems). The reaction mixture was then incubated under the following conditions: 10 min at 25 °C, 120 min at 37 °C, 5 min at 85 °C (to inactivate the reaction), and then held at 4 °C. To ensure absence of genomic DNA contamination of the isolated RNA, a negative control was included by omitting the reverse transcriptase enzyme from the reaction. Furthermore, a ‘no template’ control (NTC) reaction was performed that included all components except for the cDNA template, which was then substituted with water. The final products were stored at −20 °C before being used for qPCR.

Real-time qPCRs were performed in a 20 µl reaction mixture containing 5.42 ng cDNA, 1 x TaqMan Fast Advanced Master Mix (Thermo Fisher Scientific), and 1 x Taqman probe and primer for each gene of interest (Thermo Fisher Scientific). To test the specificity of the PCR conditions, cDNA from human whole brain (TaKaRa Bio Europe) was used as a positive control. TATA-binding protein (TBP) mRNA was used as the endogenous internal control gene, as it has been shown to be a stable and reliable reference gene in unselected leukocytes [27], and has previously been used as reference for qPCR of GABA_A_R subunit mRNA in human PBMCs [23]. TBP probes were labelled with the fluorochrome dye VIC and GABA_A_ subunit probes with FAM.

We chose to quantify mRNA for the following GABA_A_R subunits: α1, α4, α5, β2, β3, γ2 and δ. These were chosen as they have been shown to be expressed in PBMCs [22, 23], GABA_A_R incorporating these subunits are sensitive to modulation by neurosteroids [28], are modulated by mental health status [8, 9, 28], or have been previously implicated in the pathophysiology of PMDD (α4, δ) [29, 30]. See Table S1 for the corresponding TaqMan assays (ThermoFisher Scientific).

Amplification was performed in 96-well blocks using the QuantStudio 6 Flex System (Thermo Fisher Scientific) with initial denaturation at 95 °C for 20 s, followed by 45 cycles of denaturation at 95 °C for 1 s, and annealing and extension at 60 °C for 20 s. Ct values were determined by software supplied by the QuantStudio system. Ct values of each GABA_A_ subunit mRNA were normalized against the TBP internal reference gene, using the comparative Ct (2^-ΔCt^) method [31].

### Progesterone and neurosteroid quantification

Peripheral progesterone levels were quantified using chemiluminescent immunoassays by the central hospital laboratory at Norrland University Hospital, Umeå, Sweden. Detectable antibody-hapten complexes were prepared by incubating samples with progesterone-specific antibodies, streptavidin-coated microparticles and a ruthenium complex-marked derivate. The detection limit for serum progesterone was 0.05 ng/ml.

ALLO and ISO samples were quantified using ultra-high performance liquid chromatography mass spectrometry (UPLC-MS/MS) by LabLytica, Uppsala, Sweden. Serum samples were first extracted using liquid-liquid extraction in a hexane/ether solvent phase and then derivatized using 3-aminooxypropyl (trimethyl) ammonium bromide, to improve selectivity and sensitivity of detection. The lower limit of quantification (LLOQ) was 0.2 nM for ALLO and 0.1 nM for ISO.

### Experimental paradigm (fMRI task)

During the fMRI session, participants performed an emotional discrimination task, which is known to reliable engage the amygdala [32]. The task consisted of matching angry or sad faces (emotion condition) and vertical or horizontal ellipses (sensorimotor control condition). The emotional context and sex of the faces presented were balanced across trials. The paradigm was a block design, including four blocks of faces (24 trials) and five blocks of shapes (30 trials).

### MRI acquisition and preprocessing

MRI images were acquired using a 3.0 T Discovery MR750 (General Electric, Madison, WI, USA) scanner available through the Umeå Center for Functional Brain Imaging (UFBI). Acquisition parameters for T1-weighted, and blood-oxygen level dependent (BOLD) images are described previously [21].

Preprocessing of fMRI data was performed using the FSL *FMRI Expert Analysis Tool* (FEAT) [33], and included motion correction by volume-wise rigid body transformation to the middle volume, correction for magnetic field inhomogeneities, slice timing correction, spatial smoothing with a 5-mm full-width at half maximum (FWHM) Gaussian kernel, high-pass (cut-off = 100 s) temporal filtering and spatial normalization into Montreal Neurological Institute (MNI) space by affine (12 DOF) and non-linear registration (warp-resolution 8 mm).

First-level (subject-level) statistics were obtained using a general lineal model (GLM) for the contrast of interest [*Faces* > *Shapes*]. Regressors of no interest were motion parameters estimated from the spatial realignment and frames corrupted by large movements detected by FSL’s Motion Outliers tool (default metric = refrms).

### Statistical analyses

#### GABA_A_R subunit mRNA expression

Statistical analyses were performed using R [34] and the rstatix package [35]. Analyses were restricted to GABA_A_R subunits that were expressed in at least 2/3 technical replicates in most (>50%) participants. Two-way repeated measures ANCOVAs were conducted to detect *Group*, *Phase* and *Group x Phase* interaction effects, while controlling for psychiatric history, which tended to differ between the groups (see Results). We chose to control for psychiatric history as depression has previously been associated with altered GABA_A_R subunit mRNA expression in PBMCs of pregnant women [23]. Post-hoc pairwise t-tests were used to assess the direction of effects and resultant *p*-values were adjusted using the Bonferroni method. Initially, ANOVA assumptions of normality and heterogeneity of variance were checked using quantile-quantile (QQ)-plots, Shapiro-Wilk’s test and Levene’s test (means and standard deviations of 2^-ΔCt^ values for each tested subunit are provided in Table S4). Outliers were identified using the boxplot method and were classed as extreme if values were above or below 3 times the interquartile range (IQR). In all subunits investigated, extreme outliers skewed the data: we therefore opted to present ANOVA results after addressing outliers in two different ways – log-transforming data to make it conform to a more normal distribution or excluding extreme outliers. We also present distribution of the untreated data using boxplots.

#### Correlations to steroids and symptoms

Non-parametric spearman correlations were used to determine whether luteal-phase concentrations of ISO, ALLO and the ratio ISO/ALLO were related to differentially expressed GABA_A_R subunits. The variable ISO/ALLO was log-transformed to minimize problems related to the asymmetry of ratios [36]. In addition, in PMDD women, differentially expressed GABA_A_R subunits were correlated to DRSP symptom ratings obtained during menstrual cycles screened prior to inclusion. Ratings given during days −8–−1 were averaged across all screened cycles. DRSP measures assessed were total symptom score (mean of items 1 to 21 in the DRSP), depression score (mean of items 1–3), anxiety score (item 4), emotional lability score (mean of items 5 and 6) and irritability score (mean of items 7 and 8). Correlation tests of steroid and symptom measures, respectively, were corrected for multiple comparisons using FDR. Results were considered significant if *p*_*adj*_ < 0.05.

#### Relationship to fMRI measure

Mean BOLD parameter estimates were extracted from a region-of-interest (ROI) that included the right basolateral amygdala and a portion of the right parahippocampal gyrus. The ROI is derived from an analysis presented in our previous paper [21], which showed that task-related activation in this region exhibited an altered relationship to ISO/ALLO levels between PMDD women and controls during the luteal phase. Given that ALLO targets GABA_A_Rs, and that receptor composition modulates its pharmacodynamic properties [9], we wanted to examine whether differential expression of GABA_A_R subunits also related to activity in this specific region. Thus, for differentially expressed GABA_A_R subunits, women with PMDD were stratified into two equal groups with *high* or *low* luteal phase levels of the subunit. *High* and *low* groups were compared using Wilcoxon rank t-tests based on estimates of amygdala activation during the luteal phase.

## Results

### Background characteristics

Both the PMDD and control groups mainly consisted of young (mean_PMDD/Control_ = 28 years) women without children (PMDD = 69% nulliparous, Control = 74% nulliparous). There were no significant differences in age, body mass index, menstrual cycle length or parity between the groups (Table S2). Psychiatric history tended to differ between groups (*p* = 0.10): 31% of PMDD but only 11% of control women reported past depressive episodes. Progesterone and neurosteroid levels did not differ between groups across the menstrual cycle. Details of participants characteristics can be found in [21].

### Expression of GABA_A_R subunit mRNA in PBMCs

**Fig. 1:**
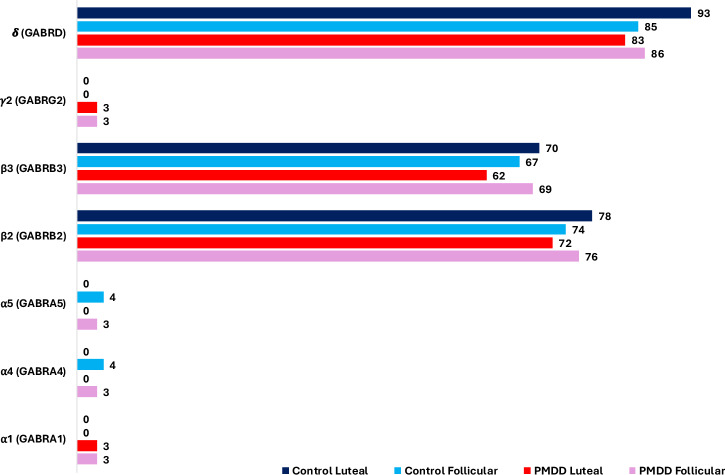
Number of individuals expressing mRNA for GABA_A_ receptor subunits across the menstrual cycle. Number of individuals per group and menstrual cycle phase having detectable mRNA levels of selected GABA_A_ receptor subunits in PBMCs harvested from venous blood samples. To be included, mRNA had to be detected reliably in at least 2/3 technical replicates. GABA_A_, γ-amino butyric acid type A; mRNA messenger ribonucleic acid, PBMC peripheral blood mononuclear cells.

The δ, β2 and β3 subunits were detected in at least 2/3 technical replicates in most women (Fig. 1, Table S3). The δ subunit was the most reliable and detected in 87% of samples across the menstrual cycle. The β2 and β3 subunits were measured in 75 and 67% of samples, respectively. Conversely, mRNA of α1, α4, α5 and γ2 subunits were undetectable in the majority of samples investigated. Chi Square tests confirmed that there were no significant differences in detection rates between groups in either phase of the menstrual cycle.

### Differential expression of GABA_A_R subunit mRNA across the menstrual cycle in PMDD and control women

**Fig. 2:**
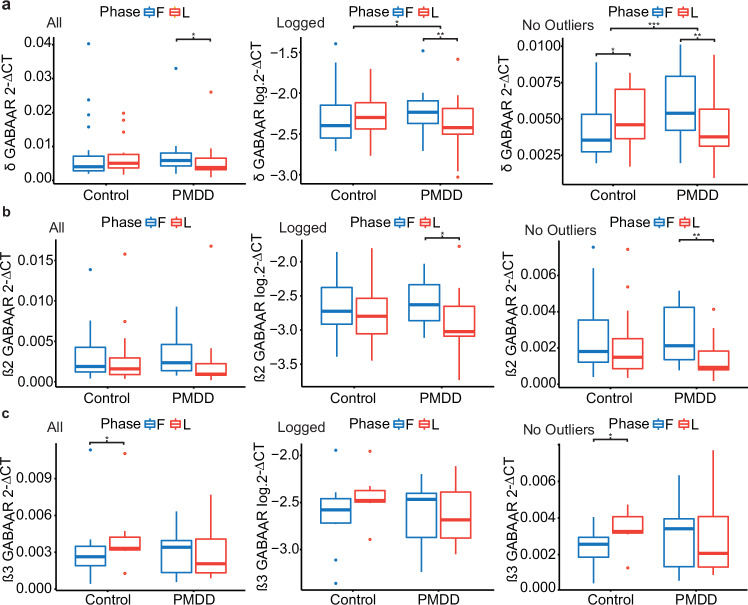
Differential expression of GABA_A_R mRNA subunit expression across the menstrual cycle in PMDD patients and controls. mRNA expression of δ, β2 and β3 subunits of the GABA_A_R subunit was quantified using RT-qPCR across the menstrual cycle in women with PMDD and asymptomatic controls. To compare change in mRNA expression between groups and across the menstrual cycle, Ct values obtained for each GABA_A_ subunit mRNA were normalized against TBP, an internal reference gene, using the comparative Ct (2^-ΔCt^) method [31]. 2^-ΔCt^ values were compared using a 2-by-2 mixed ANCOVA design, which controlled for psychiatric history. Data are illustrated with boxplots, where whiskers represent ± 1.5IQR. In each row (a, b, c corresponding to δ, β2 and β3), boxplots on the left illustrate raw data, and the middle and right plots show two alternative ways of handling outliers: using log-transformed values (middle), and removal of extreme outliers ± 3IQR (left). **a** Significant group-by-phase effects were detected for δ mRNA expression (ANCOVA1, log-transformed values: F(1,40) = 5.67, *p* = 0.02; ANCOVA2, five extreme outliers removed [4 controls, 1 PMDD]: F(1,35) = 14, *p*_*adj*_ < 0.001). Women with PMDD had decreased δ mRNA expression in the luteal compared to the follicular phase (raw, *p*_*adj*_ = 0.049; log-transformed values, *p*_*adj*_ = 0.004; 2 extreme outliers removed [1 PMDD, 1 control], *p*_*adj*_ = 0.005). **b** There was a significant main effect of menstrual cycle phase on β3 expression (ANCOVA1, log-transformed values: *F*(1,32) = 4.377, *p* = 0.04, η^2^ = 0.034). The effect was driven by women with PMDD, who had lower β2 mRNA expression in the luteal, compared to the follicular phase (log-transformed values, *p*_*adj*_ = 0.011; 2 extreme outliers removed [1 PMDD, 1 control], *p*_*adj*_ = 0.006). **c** ANCOVA effects were non-significant for the expression of β3 mRNA. However, an increased expression of β3 mRNA in the luteal compared to the follicular phase in control women was detected using within-group pairwise comparisons (raw: *p*_*adj*_ = 0.046; 1 extreme outliers removed [1 control]): *p*_*adj*_ = 0.035). CT cycle threshold, mRNA messenger ribonucleic acid, IQR interquartile range, GABA_A_R γ-aminobutyric acid type A receptor, PMDD premenstrual dysphoric disorder, RT-qPCR real-time quantitative polymerase chain reaction, TBP TATA-binding protein.

There was a significant group by phase interaction effect on the expression of δ subunit mRNA (ANCOVA with log-transformed 2^-ΔCt^ values: *F*(1,40 = 5.67, *p* = 0.02, η^2^ = 0.02) (Fig. 2a). The interaction effect was driven by a decrease in δ mRNA during the luteal phase, compared to the follicular phase (*p*_*adj*_ = 0.004), in women with PMDD. The interaction effect remained significant in all following situations: with or without controlling for psychiatric history, whether 2^-ΔCt^ values were log-transformed, or whether extreme outliers were removed outright from the analysis prior to being input into the ANOVA model.

Findings were similar for the β2 subunit (Fig. 2b). A significant phase effect (ANCOVA on log-transformed values: *F*(1,32) = 4.377, *p* = 0.04, η^2^ = 0.034) was observed, which was driven by women with PMDD exhibiting lower β2 mRNA expression in the luteal, compared to the follicular phase (*p*_*adj*_ = 0.01). Results were not significantly influenced by psychiatric history.

No significant effects of group, phase or interactions effects were detected on β3 subunit mRNA expression (Fig. 2c). However, pairwise comparisons detected a phase difference in the control group, whereby β3 mRNA expression was increased in the luteal, compared to the follicular phase (*p*_*adj*_ = 0.04).

### No relationship between neurosteroid levels, symptoms, and GABA_A_R subunit mRNA expression

No significant correlations were found between ALLO, ISO or ISO/ALLO and mRNA expression of the δ, β2 or β3 subunits in the luteal phase in either group. In addition, δ, β2 or β3 subunits expression was unrelated to total premenstrual symptoms, or core mood symptoms.

### Reduced expression of δ subunit mRNA is associated with higher amygdala activity

**Fig. 3:**
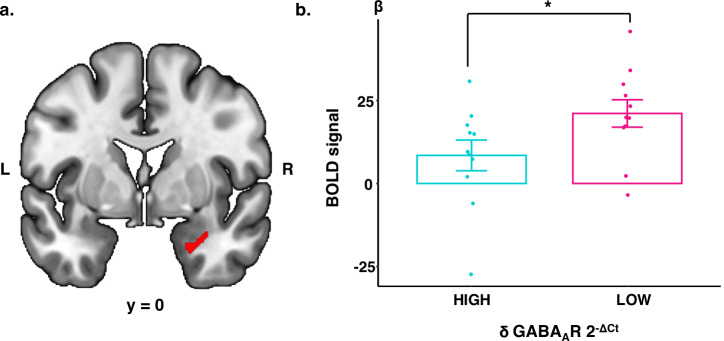
Lower mRNA expression of δ GABAAR subunits in PBMCs are related to higher amygdala activation in the luteal phase in PMDD. Emotion-related BOLD-fMRI responses in the amygdala were compared in PMDD women with high vs. low mRNA expression of δ GABA_A_R subunits in PBMCs during the luteal phase. **a** Brain slice showing the region-of-interest in the amygdala from which mean parameter estimates of BOLD-signal were extracted. Activation of this region during an emotional task has previously been shown to be differentially related to ISO/ALLO levels in the luteal phase between PMDD patients and controls [21]. **b** PMDD women with lower mRNA expression of δ GABA_A_R subunits had higher emotion-related activation of the right amygdala during the luteal phase, compared with women with higher expression (*p* = 0.04). ALLO allopregnanolone, BOLD-fMRI blood-oxygen level dependent functional magnetic imaging, CT cycle threshold, ISO isoallopregnanolone, mRNA messenger ribonucleic acid, GABA_A_R γ-aminobutyric acid type A receptor, L left, PBMC peripheral blood mononuclear cell, PMDD premenstrual dysphoric disorder, R right.

Women with PMDD with lower mRNA expression of the δ subunit in PBMCs had higher emotion-related amygdala activity during the luteal phase, compared with women with higher expression of the subunit (*p* = 0.04) (Fig. 3). Amygdala activation was not related to high or low mRNA expression of β2 or β3 subunits.

## Discussion

The present study provides the first evidence of differential GABA_A_R subunit gene expression across the menstrual cycle in women with PMDD. Specifically, δ and β2 subunit mRNAs were decreased in PBMCs in the luteal phase compared to the follicular phase of women with PMDD. Furthermore, lower δ subunit mRNA expression in PMDD was related to increased amygdala activation during an emotional task (compared to a sensorimotor control task) during the luteal phase. Our findings suggest that altered GABA_A_R plasticity in PMDD mediates altered reactivity of brain regions involved in emotion generation, which might explain the characteristic mood symptoms of the disorder.

GABA_A_R containing δ subunits mediate tonic (persistent) inhibition and are thus thought to be key modulators of neuronal excitability in the CNS [6, 37]. In addition, δ-containing GABA_A_R are particularly sensitive to positive allosteric modulation by neurosteroids such as ALLO [6, 7, 38]. Some have even argued that δ-containing GABA_A_R are the preferred, if not the only, site of action for neurosteroids in the physiological concentration range [39–41]. Rodent studies have shown that δ subunit expression in the brain is regulated by reproductive events [12, 13, 16]. Regulatory effects seem to be directly mediated by ALLO (and not progesterone), as regulation of the δ subunit can be inhibited by application of finasteride (which blocks the conversion of progesterone to ALLO) and is unaffected by mifepristone (a progesterone receptor antagonist) [13]. Studies have found that down-regulation of δ-GABA_A_Rs occurs in the hippocampus and amygdala in the presence of chronically high ALLO levels, while up-regulation is seen upon steroid withdrawal [12, 13, 16]. Thus, δ-GABA_A_Rs are under constant dynamic modulation across the ovarian cycle, and deficits in this system (such as in δ−/δ− or δ+/δ− knock-out mice) are associated with increased neuronal excitability and anxiety [12, 13]. Our finding of reduced δ subunit expression in women with PMDD may indicate an impaired up-regulation of δ-GABA_A_Rs in the context of ALLO-withdrawal in the late luteal phase. This is further supported by studies of GABA_A_R sensitivity in PMDD showing that PMDD women are less sensitive to low doses of ethanol and pregnanolone (the 5β-pregnane epimer of ALLO) in the late luteal compared to the mid-follicular phase [19, 42]. Both ethanol and pregnanolone are thought to be particularly potent modulators of δ-containing GABA_A_Rs [43, 44]. In contrast, women with PMDD have previously been shown to be more sensitive to supraphysiological doses of ALLO in the luteal phase [45]. However, supraphysiological doses of ALLO also activate synaptic γ2-containing GABA_A_Rs [7, 37], and differences in sensitivity between patients and controls in this context may thus be related to differential modulation of other GABA_A_Rs subtypes, which were unfortunately not detectable in the present study. Moreover, altered ALLO sensitivity in PMDD may be influenced by other factors that were not investigated in the present study. First, lower cerebral GABA levels in the luteal phase have been reported in women with PMDD [46]. Since neurosteroid efficacy at the δ GABA_A_R is dependent on ambient GABA concentrations [47], lower GABA levels may mediate a lower sensitivity to neurosteroids. Second, δ GABA_A_R tone may be influenced by altered neurosteroid metabolism and synthesis in the brain. While this has never been investigated in PMDD, reduced levels of ALLO in the cerebrospinal fluid is found in conditions with overlapping symptomatology, including depression, anxiety, and chronic stress [48, 49]. Nonetheless, findings from studies of progesterone/ALLO withdrawal in δ-GABA_A_R-deficient rodents provides an interesting parallel with PMDD [12, 13]. Inefficient regulation of δ-GABA_A_Rs in response to ALLO fluctuations may thus be an important mechanism underlying the susceptibility of PMDD women to experience severe symptoms in response to ovarian hormone fluctuations.

Remarkably, we found that lower δ subunit expression was associated with increased activity in the basolateral amygdala. This is particularly pertinent to explaining the mood symptoms of PMDD as the amygdala is thought to signal salience and novelty of stimuli and participates in the generation of emotion [50–52]. Furthermore, amygdala hyperactivity has been found to be a transdiagnostic feature of multiple psychiatric disorders, including anxiety and depressive disorders [53], and is thought to mediate exaggerated and inappropriate reactivity to stimuli [54]. One of the most consistent findings in neuroimaging studies of PMDD is that amygdala activity is increased in the luteal phase [55]. One early fMRI study, albeit in healthy women, could show that exogenous administration of progesterone resulting in luteal-phase ALLO levels could selectively enhance amygdala activity in response to emotional stimuli [56]. In addition, we have previously shown that activity in the basolateral amygdala is differentially associated with ISO/ALLO levels in PMDD, as compared to control women [21]. δ-GABA_A_Rs are expressed in the amygdala, although their precise location seems to be species-specific. Indeed, they are found most abundantly in the intercalated cell masses in mouse [57] and in the basolateral amygdala in the rhesus monkey [58]. Histochemical stains of δ subunits in the human brain are not available. Nonetheless, a study in mice showed that ALLO influences neural activity in the basolateral amygdala, and that this effect was mediated by δ-GABA_A_Rs [59]. In the same study, ALLO-induced modulation of the basolateral amygdala related to anxiety expression [59]. The amygdala may thus be a key region mediating ALLO’s modulatory effects on mood, through its actions on δ-GABA_A_Rs. However, it is important to note that δ-GABA_A_R expression has been found to be altered in conditions of stress, psychiatric disorders, alcohol abuse, and reproductive events in other regions of the brain, including frontal regions and hippocampus [9, 13, 40, 60]. Further investigations should attempt to determine the behavior consequences of altered expression of δ-GABA_A_R in other brain regions, and in relation to other important modulators such as stress, to gain a better understanding of the neuropsychopathology of PMDD.

The significance of differential β subunit expression in PMDD is not clear. The isoform of the β subunit is not thought to affect neurosteroid modulation of GABA_A_R function [7, 61]. However, functional GABA_A_Rs require the inclusion of β subunits, and β3 seems to be particularly important for inhibitory transmission - at least in the hippocampus - as evidenced by significant reductions in inhibitory currents following knock-out of β3 in mice, but not deletion of β1 or β2 [62]. Furthermore, neurosteroid-dependent phosphorylation of the β3 subunit via membrane progesterone receptors promotes GABA_A_R insertion in the cell membrane leading to increased tonic inhibition [63, 64]. In addition, the β3 subunit has been demonstrated to be strictly required for spontaneous gating of the GABA_A_R, which results in neuronal inhibitory currents independent of GABA [65]. Higher β3 expression in the luteal phase in controls may thus contribute to reduced neuronal excitability in these women. Conversely, an altered proportion of β2/β3 subunit in GABA_A_Rs in PMDD indicates further abnormalities in GABA_A_R plasticity, which possibly confer reduced GABAergic tone in the luteal phase.

Our findings must be considered in light of the following limitations. The first, and most important, is that we base our discussion about GABA_A_R subunit expression on measures of mRNA levels in PBMCs. However, a myriad of posttranslational mechanisms contribute to poor observed correspondence between mRNA levels and functional proteins [66]. This might also explain why we did not find significant relationships between serum levels of ALLO and GABA_A_R subunits. However, genes that are differentially expressed (in our case between menstrual cycle phases, and between patients and control) may correlate better to their protein product [66]. Nonetheless, studies which also examine GABA_A_R subunit protein expression are necessary to determine whether subunits are truly differentially expressed in PMDD. A second limitation is that we do not know whether GABA_A_R subunit mRNAs in PBMCs is related to expression in the brain. However, altered gene expression in PBMCs has been shown to reflect brain changes following maternal separation in rodents [67]. In humans, PBMC gene expression (amongst which, genes coding for GABA_A_ receptor subunits) has been used to successfully predict onset and progression of PTSD in trauma-survivors [68]. Changes in PBMC gene expression may thus provide some clues about transcriptional changes in the brain. A third limitation is that mRNA levels of GABA_A_R subunits in our samples were low, and many PCR cycles (>35) were required for detection. This meant that standard deviations of Ct values among replicates were relatively high, and for most subunits, mRNA levels were undetectable. The variability of our data is problematic and may have influenced our results. Lastly, mood symptom ratings were averaged from screening cycles and not collected during actual scanning days. This might have influenced our null finding in relating symptom severity to GABA_A_R subunit expression.

In conclusion, our study is the first quantification of gene expression of GABA_A_R subunits in women with PMDD. We found evidence of reduced δ subunit expression in PMDD women, which was related to increased reactivity to emotional stimuli in the amygdala. Abnormal regulation of δ-containing GABA_A_R by ALLO in the amygdala might be an important pathophysiological mechanism by which symptoms in the luteal phase are elicited. Pharmacological studies using drugs which specifically target δ GABA_A_R receptors may provide interesting insights into how modulation of this receptor correlates to behavioral change in PMDD.

## Supplementary information

Supplementary Materials

## Data Availability

Data is available upon request to authors.

## References

[1] Halbreich U, Borenstein J, Pearlstein T, Kahn LS. The prevalence, impairment, impact, and burden of premenstrual dysphoric disorder (PMS/PMDD). Psychoneuroendocrinology. 2003;28(Suppl 3):1–23. 12892987 10.1016/s0306-4530(03)00098-2

[2] Reilly TJ, Patel S, Unachukwu IC, Knox C-L, Wilson CA, Craig MC, et al. The prevalence of premenstrual dysphoric disorder: systematic review and meta-analysis. J Affect Disord. 2024;349:534–40. 38199397 10.1016/j.jad.2024.01.066

[3] Bäckström T, Bixo M, Johansson M, Nyberg S, Ossewaarde L, Ragagnin G, et al. Allopregnanolone and mood disorders. Prog Neurobiol. 2014;113:88–94. 23978486 10.1016/j.pneurobio.2013.07.005

[4] Bäckström T, Haage D, Löfgren M, Johansson IM, Strömberg J, Nyberg S, et al. Paradoxical effects of GABA-A modulators may explain sex steroid induced negative mood symptoms in some persons. Neuroscience. 2011;191:46–54. 21600269 10.1016/j.neuroscience.2011.03.061

[5] Chua HC, Chebib M. GABA(A) receptors and the diversity in their structure and pharmacology. Adv Pharmacol. 2017;79:1–34. 28528665 10.1016/bs.apha.2017.03.003

[6] Farrant M, Nusser Z. Variations on an inhibitory theme: phasic and tonic activation of GABA(A) receptors. Nat Rev Neurosci. 2005;6:215–29. 15738957 10.1038/nrn1625

[7] Belelli D, Casula A, Ling A, Lambert JJ. The influence of subunit composition on the interaction of neurosteroids with GABA(A) receptors. Neuropharmacology. 2002;43:651–61. 12367610 10.1016/s0028-3908(02)00172-7

[8] Locci A, Geoffroy P, Miesch M, Mensah-Nyagan AG, Pinna G. Social isolation in early versus late adolescent mice is associated with persistent behavioral deficits that can be improved by neurosteroid-based treatment. Front Cell Neurosci. 2017;11:208. 28900387 10.3389/fncel.2017.00208 PMC5581875

[9] Locci A, Pinna G. Neurosteroid biosynthesis down-regulation and changes in GABA. Br J Pharmacol. 2017;174:3226–41. 28456011 10.1111/bph.13843 PMC5595768

[10] Yu R, Follesa P, Ticku MK. Down-regulation of the GABA receptor subunits mRNA levels in mammalian cultured cortical neurons following chronic neurosteroid treatment. Brain Res Mol Brain Res. 1996;41:163–8. 8883948 10.1016/0169-328x(96)00087-3

[11] Gulinello M, Gong QH, Smith SS. Progesterone withdrawal increases the alpha4 subunit of the GABA(A) receptor in male rats in association with anxiety and altered pharmacology - a comparison with female rats. Neuropharmacology. 2002;43:701–14. 12367616 10.1016/s0028-3908(02)00171-5 PMC2887344

[12] Maguire JL, Stell BM, Rafizadeh M, Mody I. Ovarian cycle-linked changes in GABA(A) receptors mediating tonic inhibition alter seizure susceptibility and anxiety. Nat Neurosci. 2005;8:797–804. 15895085 10.1038/nn1469

[13] Maguire J, Mody I. Neurosteroid synthesis-mediated regulation of GABA(A) receptors: relevance to the ovarian cycle and stress. J Neurosci. 2007;27:2155–62. 17329412 10.1523/JNEUROSCI.4945-06.2007 PMC6673487

[14] Gulinello M, Orman R, Smith SS. Sex differences in anxiety, sensorimotor gating and expression of the alpha4 subunit of the GABAA receptor in the amygdala after progesterone withdrawal. Eur J Neurosci. 2003;17:641–8. 12581182 10.1046/j.1460-9568.2003.02479.x PMC2887345

[15] Shen H, Gong QH, Yuan M, Smith SS. Short-term steroid treatment increases delta GABAA receptor subunit expression in rat CA1 hippocampus: pharmacological and behavioral effects. Neuropharmacology. 2005;49:573–86. 15950994 10.1016/j.neuropharm.2005.04.026 PMC2887348

[16] Maguire J, Mody I. GABA(A)R plasticity during pregnancy: relevance to postpartum depression. Neuron. 2008;59:207–13. 18667149 10.1016/j.neuron.2008.06.019 PMC2875248

[17] Sundström I, Nyberg S, Bäckström T. Patients with premenstrual syndrome have reduced sensitivity to midazolam compared to control subjects. Neuropsychopharmacology. 1997;17:370–81. 9397425 10.1016/S0893-133X(97)00086-9

[18] Sundström I, Ashbrook D, Bäckström T. Reduced benzodiazepine sensitivity in patients with premenstrual syndrome: a pilot study. Psychoneuroendocrinology. 1997;22:25–38. 9141149 10.1016/s0306-4530(96)00035-2

[19] Nyberg S, Wahlström G, Bäckström T, Sundström Poromaa I. Altered sensitivity to alcohol in the late luteal phase among patients with premenstrual dysphoric disorder. Psychoneuroendocrinology. 2004;29:767–77. 15110926 10.1016/S0306-4530(03)00121-5

[20] Timby E, Bäckström T, Nyberg S, Stenlund H, Wihlbäck AC, Bixo M. Women with premenstrual dysphoric disorder have altered sensitivity to allopregnanolone over the menstrual cycle compared to controls-a pilot study. Psychopharmacology. 2016;233:2109–17. 26960697 10.1007/s00213-016-4258-1

[21] Stiernman L, Dubol M, Comasco E, Sundström-Poromaa I, Boraxbekk CJ, Johansson M, et al. Emotion-induced brain activation across the menstrual cycle in individuals with premenstrual dysphoric disorder and associations to serum levels of progesterone-derived neurosteroids. Transl Psychiatry. 2023;13:124. 37055419 10.1038/s41398-023-02424-3 PMC10101953

[22] Alam S, Laughton DL, Walding A, Wolstenholme AJ. Human peripheral blood mononuclear cells express GABAA receptor subunits. Mol Immunol. 2006;43:1432–42. 16213022 10.1016/j.molimm.2005.07.025

[23] Bhandage AK, Hellgren C, Jin Z, Olafsson EB, Sundström-Poromaa I, Birnir B. Expression of GABA receptors subunits in peripheral blood mononuclear cells is gender dependent, altered in pregnancy and modified by mental health. Acta Physiol. 2015;213:575–85. 10.1111/apha.12440 25529063

[24] Sheehan DV, Lecrubier Y, Sheehan KH, Amorim P, Janavs J, Weiller E, et al. The Mini-International Neuropsychiatric Interview (M.I.N.I.): the development and validation of a structured diagnostic psychiatric interview for DSM-IV and ICD-10. J Clin Psychiatry. 1998;59(Suppl 20):22–33. 9881538

[25] Endicott J, Nee J, Harrison W. Daily Record of Severity of Problems (DRSP): reliability and validity. Arch Womens Ment Health. 2006;9:41–9. 16172836 10.1007/s00737-005-0103-y

[26] Sundström-Poromaa I, Comasco E, Sumner R, Luders E. Progesterone - friend or foe? Front Neuroendocrinol. 2020;59:100856. 32730861 10.1016/j.yfrne.2020.100856

[27] Ledderose C, Heyn J, Limbeck E, Kreth S. Selection of reliable reference genes for quantitative real-time PCR in human T cells and neutrophils. BMC Res Notes. 2011;4:427. 22011438 10.1186/1756-0500-4-427 PMC3229292

[28] Mitchell EA, Herd MB, Gunn BG, Lambert JJ, Belelli D. Neurosteroid modulation of GABAA receptors: molecular determinants and significance in health and disease. Neurochem Int. 2008;52:588–95. 18055067 10.1016/j.neuint.2007.10.007

[29] Smith SS, Ruderman Y, Frye C, Homanics G, Yuan M. Steroid withdrawal in the mouse results in anxiogenic effects of 3alpha,5beta-THP: a possible model of premenstrual dysphoric disorder. Psychopharmacology. 2006;186:323–33. 16193334 10.1007/s00213-005-0168-3 PMC2887339

[30] Hantsoo L, Epperson CN. Allopregnanolone in premenstrual dysphoric disorder (PMDD): evidence for dysregulated sensitivity to GABA-A receptor modulating neuroactive steroids across the menstrual cycle. Neurobiol Stress. 2020;12:100213. 32435664 10.1016/j.ynstr.2020.100213 PMC7231988

[31] Schmittgen TD, Livak KJ. Analyzing real-time PCR data by the comparative C(T) method. Nat Protoc. 2008;3:1101–8. 18546601 10.1038/nprot.2008.73

[32] Hariri AR, Tessitore A, Mattay VS, Fera F, Weinberger DR. The amygdala response to emotional stimuli: a comparison of faces and scenes. Neuroimage. 2002;17:317–23. 12482086 10.1006/nimg.2002.1179

[33] Jenkinson M, Beckmann CF, Behrens TE, Woolrich MW, Smith SM. FSL. Neuroimage. 2012;62:782–90. 21979382 10.1016/j.neuroimage.2011.09.015

[34] Team Rc. R: A language and environment for statistical computing. Vienna, Austria: R Foundation for Statistical Computing; 2021.

[35] Kassambara A rstatix: Pipe-friendly framework for basic statistical tests. 2021. https://rpkgs.datanovia.com/rstatix/

[36] Sollberger S, Ehlert U. How to use and interpret hormone ratios. Psychoneuroendocrinology. 2016;63:385–97. 26521052 10.1016/j.psyneuen.2015.09.031

[37] Zheleznova NN, Sedelnikova A, Weiss DS. Function and modulation of delta-containing GABA(A) receptors. Psychoneuroendocrinology. 2009;34(Suppl 1):S67–73. 19766404 10.1016/j.psyneuen.2009.08.010 PMC2794972

[38] Wohlfarth KM, Bianchi MT, Macdonald RL. Enhanced neurosteroid potentiation of ternary GABAAReceptors containing the δ subunit. J Neurosci. 2002;22:1541–9. 11880484 10.1523/JNEUROSCI.22-05-01541.2002 PMC6758857

[39] Belelli D, Lambert JJ. Neurosteroids: endogenous regulators of the GABA(A) receptor. Nat Rev Neurosci. 2005;6:565–75. 15959466 10.1038/nrn1703

[40] Maguire J, Ferando I, Simonsen C, Mody I. Excitability changes related to GABAA receptor plasticity during pregnancy. J Neurosci. 2009;29:9592–601. 19641122 10.1523/JNEUROSCI.2162-09.2009 PMC2875247

[41] Stell BM, Brickley SG, Tang CY, Farrant M, Mody I. Neuroactive steroids reduce neuronal excitability by selectively enhancing tonic inhibition mediated by delta subunit-containing GABAA receptors. Proc Natl Acad Sci USA. 2003;100:14439–44. 14623958 10.1073/pnas.2435457100 PMC283610

[42] Sundström I, Andersson A, Nyberg S, Ashbrook D, Purdy RH, Bäckström T. Patients with premenstrual syndrome have a different sensitivity to a neuroactive steroid during the menstrual cycle compared to control subjects. Neuroendocrinology. 1998;67:126–38. 9508043 10.1159/000054307

[43] Wallner M, Hanchar HJ, Olsen RW. Low dose acute alcohol effects on GABA A receptor subtypes. Pharmacol Ther. 2006;112:513–28. 16814864 10.1016/j.pharmthera.2006.05.004 PMC2847605

[44] Lagrange A. Dancing the delta shuffle: neurosteroids regulate GABAA receptor expression. Epilepsy Curr. 2006;6:14–7. 16477317 10.1111/j.1535-7511.2005.00081.x PMC1363372

[45] Timby E, Balgård M, Nyberg S, Spigset O, Andersson A, Porankiewicz-Asplund J, et al. Pharmacokinetic and behavioral effects of allopregnanolone in healthy women. Psychopharmacology. 2006;186:414–24. 16177884 10.1007/s00213-005-0148-7

[46] Liu B, Wang G, Gao D, Gao F, Zhao B, Qiao M, et al. Alterations of GABA and glutamate-glutamine levels in premenstrual dysphoric disorder: a 3T proton magnetic resonance spectroscopy study. Psychiatry Res. 2015;231:64–70. 25465316 10.1016/j.pscychresns.2014.10.020

[47] Houston CM, McGee TP, Mackenzie G, Troyano-Cuturi K, Rodriguez PM, Kutsarova E, et al. Are extrasynaptic GABAA receptors important targets for sedative/hypnotic drugs? J Neurosci. 2012;32:3887–97. 22423109 10.1523/JNEUROSCI.5406-11.2012 PMC4620914

[48] Maguire J. Stress-induced plasticity of GABAergic inhibition. Front Cell Neurosci. 2014;8:157. 24936173 10.3389/fncel.2014.00157 PMC4047962

[49] Schüle C, Nothdurfter C, Rupprecht R. The role of allopregnanolone in depression and anxiety. Prog Neurobiol. 2014;113:79–87. 24215796 10.1016/j.pneurobio.2013.09.003

[50] Santos A, Mier D, Kirsch P, Meyer-Lindenberg A. Evidence for a general face salience signal in human amygdala. NeuroImage. 2011;54:3111–6. 21081170 10.1016/j.neuroimage.2010.11.024

[51] Sander D, Grafman J, Zalla T. The human amygdala: an evolved system for relevance detection. Rev Neurosci. 2003;14:303–16. 14640318 10.1515/revneuro.2003.14.4.303

[52] Morawetz C, Riedel MC, Salo T, Berboth S, Eickhoff SB, Laird AR, et al. Multiple large-scale neural networks underlying emotion regulation. Neurosci Biobehav Rev. 2020;116:382–95. 32659287 10.1016/j.neubiorev.2020.07.001

[53] McTeague LM, Rosenberg BM, Lopez JW, Carreon DM, Huemer J, Jiang Y, et al. Identification of common neural circuit disruptions in emotional processing across psychiatric disorders. Am J Psychiatry. 2020;177:411–21. 31964160 10.1176/appi.ajp.2019.18111271 PMC7280468

[54] Menon V. Large-scale brain networks and psychopathology: a unifying triple network model. Trends Cogn Sci. 2011;15:483–506. 21908230 10.1016/j.tics.2011.08.003

[55] Dubol M, Epperson CN, Lanzenberger R, Sundström-Poromaa I, Comasco E. Neuroimaging premenstrual dysphoric disorder: a systematic and critical review. Front Neuroendocrinol. 2020;57:100838. 32268180 10.1016/j.yfrne.2020.100838

[56] van Wingen GA, van Broekhoven F, Verkes RJ, Petersson KM, Bäckström T, Buitelaar JK, et al. Progesterone selectively increases amygdala reactivity in women. Mol Psychiatry. 2008;13:325–33. 17579609 10.1038/sj.mp.4002030

[57] Hörtnagl H, Tasan RO, Wieselthaler A, Kirchmair E, Sieghart W, Sperk G. Patterns of mRNA and protein expression for 12 GABAA receptor subunits in the mouse brain. Neuroscience. 2013;236:345–72. 23337532 10.1016/j.neuroscience.2013.01.008 PMC3605588

[58] Sperk G, Kirchmair E, Bakker J, Sieghart W, Drexel M, Kondova I. Immunohistochemical distribution of 10 GABA. J Comp Neurol. 2020;528:2551–68. 32220012 10.1002/cne.24910 PMC7496627

[59] Antonoudiou P, Colmers PLW, Walton NL, Weiss GL, Smith AC, Nguyen DP, et al. Allopregnanolone mediates affective switching through modulation of oscillatory states in the basolateral amygdala. Biol Psychiatry. 2022;91:283–93. 34561029 10.1016/j.biopsych.2021.07.017 PMC8714669

[60] Merali Z, Du L, Hrdina P, Palkovits M, Faludi G, Poulter MO, et al. Dysregulation in the suicide brain: mRNA expression of corticotropin-releasing hormone receptors and GABA(A) receptor subunits in frontal cortical brain region. J Neurosci. 2004;24:1478–85. 14960621 10.1523/JNEUROSCI.4734-03.2004 PMC6730322

[61] Wang M. Neurosteroids and GABA-A receptor function. Front Endocrinol. 2011;2:44. 10.3389/fendo.2011.00044 PMC3356040 22654809

[62] Nguyen QA, Nicoll RA. The GABA. Neuron. 2018;98:718–25.e3. 29706582 10.1016/j.neuron.2018.03.046 PMC6089239

[63] Abramian AM, Comenencia-Ortiz E, Modgil A, Vien TN, Nakamura Y, Moore YE, et al. Neurosteroids promote phosphorylation and membrane insertion of extrasynaptic GABAA receptors. Proc Natl Acad Sci USA. 2014;111:7132–7. 24778259 10.1073/pnas.1403285111 PMC4024867

[64] Vien TN, Ackley MA, Doherty JJ, Moss SJ, Davies PA. Preventing phosphorylation of the GABA. Front Mol Neurosci. 2022;15:817996. 35431797 10.3389/fnmol.2022.817996 PMC9009507

[65] Sexton CA, Penzinger R, Mortensen M, Bright DP, Smart TG. Structural determinants and regulation of spontaneous activity in GABA. Nat Commun. 2021;12:5457. 34526505 10.1038/s41467-021-25633-0 PMC8443696

[66] Koussounadis A, Langdon SP, Um IH, Harrison DJ, Smith VA. Relationship between differentially expressed mRNA and mRNA-protein correlations in a xenograft model system. Sci Rep. 2015;5:10775. 26053859 10.1038/srep10775 PMC4459080

[67] van Heerden JH, Conesa A, Stein DJ, Montaner D, Russell V, Illing N. Parallel changes in gene expression in peripheral blood mononuclear cells and the brain after maternal separation in the mouse. BMC Res Notes. 2009;2:195. 19781058 10.1186/1756-0500-2-195 PMC2759952

[68] Segman RH, Shefi N, Goltser-Dubner T, Friedman N, Kaminski N, Shalev AY. Peripheral blood mononuclear cell gene expression profiles identify emergent post-traumatic stress disorder among trauma survivors. Mol Psychiatry. 2005;10:500–13, 425. 15685253 10.1038/sj.mp.4001636

