# Supplementary material for: Transcription of GABAA receptor subunits in circulating monocytes and association to emotional brain function in premenstrual dysphoric disorder
Source: Transl Psychiatry. 2025 Jul 23;15:255. doi: 10.1038/s41398-025-03465-6 (PMC12287299; doi:10.1038/s41398-025-03465-6)
Supplement: Supplementary file 1 — Supplementary Materials [file 41398_2025_3465_MOESM1_ESM.docx]

**Supplementary Information**

**Table S1.** TaqMan gene expression assays used for quantification of GABA_A_ receptor subunit mRNA

| Gene | Subunit | Assay ID |
| --- | --- | --- |
| GABRA1 | α1 | hs00971228_m1 |
| GABRA4 | α4 | hs00608034_m1 |
| GABRA5 | α5 | hs00181291_m1 |
| GABRB2 | β2 | hs00241451_m1 |
| GABRB3 | β3 | hs00241459_m1 |
| GABRG2 | γ2 | hs00168093_m1 |
| GABRD | δ | hs00181309_m1 |

**Table S2.** Baseline characteristics of women with PMDD and controls.

|  | PMDD (N=29)  Mean (SD) or N (%) | | Controls (N=27)  Mean (SD) or N (%) | |
| --- | --- | --- | --- | --- |
| Demographics | | | | |
| Age (years) | 28.5 (6.1) | | 28.3 (5.7) | |
| BMI | 23.7 (3.2) | | 24.4 (4.1) | |
| Menstrual cycle length (days) | 27.7 (1.9) | | 28.6 (1.9) | |
| Psychiatric history | 9 (31.0) | | 3 (11.1) | |
| *Depression* | 8 (27.6) | | 3 (11.1) | |
| *Eating disorder* | 1 (3.45) | | 0 | |
| Parous | 9 (31.0) | | 7 (25.9) | |
| DRSP ratings | *Mid-follicular* | *Late-luteal* | *Mid-follicular* | *Late-luteal* |
| Total symptom score | 25.5 (3.5) | 56.7 (14.3) | 24.2 (3.3) | 24.4 (3.0) ***** |
| Depression score | 3.5 (0.6) | 8.3 (2.8) | 3.4 (0.7) | 3.5 (0.6) ***** |
| Anxiety score | 1.2 (0.3) | 2.8 (1.0) | 1.1 (0.2) | 1.1 (0.2) * |
| Emotion lability score | 2.3 (0.4) | 9.3 (3.1) | 2.2 (0.4) | 3.4 (0.4) * |
| Irritability score | 2.4 (0.4) | 5.3 (2.2) | 2.4 (0.6) | 2.9 (0.4) * |
| Steroids | *Mid-follicular*  **Mean (IQR)** **or N (%)** | *Late-luteal*  **Mean (IQR) or N (%)** | *Mid-follicular*  **Mean (IQR)** **or N (%)** | *Late-luteal*  **Mean (IQR)** **or N (%)** |
| Test day | +8.0 (1.9) | -4.4 (2.0) | +7.7 (1.4) | -3.9 (1.7) |
| Progesterone (nmol/L) | 0.6 (0.5) | 23.8 (17.5) | 0.7 (0.5) | 23.4 (20.4) |
| Estradiol (pmol/L) | 309 (201) | 424 (238) | 263 (162) | 432 (156) |
| ALLO (nmol/L) | 0.337 (0.098) | 2.110 (1.420) | 0.419 (0.247) | 2.208 (1.060) |
| *Missing* | 6 (20.7) | 0 | 3 (11.1) | 2 (7.4) |
| ISO (nmol/L) | 0.127 (0.020) | 0.742 (0.484) | 0.137 (0.044) | 0.844 (0.652) |
| *Missing*^†^ | 21 (72.4) | 0 | 16 (59.3) | 3 (11.1) |
| ISO/ALLO (nmol/L) | 0.367 (0.093) | 0.349 (0.108) | 0.271 (0.079) | 0.358 (0.153) |
| *Missing* | 21 (72.4) | 0 | 16 (59.3) | 3 (11.1) |

Reproduced, with permission, from (1). Total symptom scores are the mean summed ratings for all 21 symptom items of the DRSP scale (minimum=21, maximum=126) over days +5 to +11 for the mid-follicular phase, and days -8 to -1 for the late-luteal phase. The depression scores include the DRSP items “depressed”, “hopeless” and “guilty” (minimum=3, maximum=18); anxiety scores correspond to the item “anxious” (minimum=1; maximum=6); emotion lability scores include the items “mood swings” and “easily hurt” (minimum=2; maximum=12); and irritability scores include the items “irritable” and “conflicts” (minimum=2, maximum=12). Differences between groups were assessed using Mann-Whitney U-tests for continuous variables, and Fisher’s exact tests for categorical variables. *Significant group difference at *p*<0.05. Abbreviations: ALLO, Allopregnanolone; BMI, body mass index; DRSP, Daily Record of Severity of Problems; IQR, Interquartile Range; ISO, Isoallopregnanolone; PMDD, Premenstrual Dysphoric Disorder; SD, Standard Deviation.
^†^The large proportion of missing values for serum ISO was due to the lower detection limit of the method (see Materials and Methods, Steroid analysis).

1. Stiernman L, Dubol M, Comasco E, Sundström-Poromaa I, Boraxbekk CJ, Johansson M, et al. Emotion-induced brain activation across the menstrual cycle in individuals with premenstrual dysphoric disorder and associations to serum levels of progesterone-derived neurosteroids. Transl Psychiatry. 2023;13(1):124.

**Table S3.** Frequency table over the number of individuals (%) expressing mRNA for GABA_A_ receptor subunits in PBMCs (in at least 2/3 technical replicates) across the menstrual cycle.

|  | PMDD  (N=29) | | Controls  (N=27) | | Total  (N=56) | |
| --- | --- | --- | --- | --- | --- | --- |
|  | **N (%)**  *Follicular* | **N (%)**  *Luteal* | **N (%)**  *Follicular* | **N (%)**  *Luteal* | **N (%)**  *Follicular* | **N (%)**  *Luteal* |
| α1 | 1 (3%) | 1 (3%) | 0 (0%) | 0 (0%) | 1 (2%) | 1 (2%) |
| α4 | 1 (3%) | 0 (0%) | 1 (4%) | 0 (0%) | 2 (4%) | 0 (0%) |
| α5 | 1 (3%) | 0 (0%) | 1 (4%) | 0 (0%) | 2 (4%) | 0 (0%) |
| β2 | 22 (76%) | 21 (72%) | 20 (74%) | 21 (78%) | 42 (75%) | 42 (75%) |
| β3 | 20 (69%) | 18 (62%) | 18 (67%) | 19 (70%) | 38 (68%) | 37 (66%) |
| γ2 | 1 (3%) | 1 (3%) | 0 (0%) | 0 (0%) | 1 (2%) | 1 (2%) |
| δ | 25 (86%) | 24 (83%) | 23 (85%) | 25 (93%) | 48 (86%) | 49 (88%) |

**Table S4.** Mean and standard deviations for each mRNA subunit tested (2^-ΔCt^ values) using 2-by-2 mixed ANCOVAs. Equality of variance was confirmed using Levene’s test.

|  | PMDD  (N=29) | | | | Controls  (N=27) | | | |
| --- | --- | --- | --- | --- | --- | --- | --- | --- |
|  | **Log-transformed (mean, SD)** | | **Outliers removed**  **(mean, SD)** | | **Log-transformed (mean, SD)** | | **Outliers removed**  **(mean, SD)** | |
|  | *Follicular* | *Luteal* | *Follicular* | *Luteal* | *Follicular* | *Luteal* | *Follicular* | *Luteal* |
| β2 | -2.68 (0.342) | -2.89 (0.406) | 0.003 (0.002) | 0.001 (0.001) | -2.68 (0.387) | -2.72 (0.423) | 0.003 (0.002) | 0.002 (0.002) |
| β3 | -2.59 (0.297) | -2.65 (0.282) | 0.003 (0.002) | 0.003 (0.002) | -2.64 (0.388) | -2.44 (0.229) | 0.002 (0.001) | 0.003 (0.001) |
| δ | -2.24 (0.26) | -2.40 (0.323) | 0.006 (0.002) | 0.004 (0.002) | -2.28 (0.367) | -2.28 (0.294) | 0.004 (0.002) | 0.005 (0.002) |
